# Global Analyses of Small Interfering RNAs Derived from *Bamboo mosaic virus* and Its Associated Satellite RNAs in Different Plants

**DOI:** 10.1371/journal.pone.0011928

**Published:** 2010-08-02

**Authors:** Kuan-Yu Lin, Chi-Ping Cheng, Bill Chia-Han Chang, Wei-Chi Wang, Ying-Wen Huang, Yun-Shien Lee, Hsien-Da Huang, Yau-Heiu Hsu, Na-Sheng Lin

**Affiliations:** 1 Institute of Biotechnology, National Cheng Kung University, Tainan, Taiwan; 2 Department of Life Science, Tzu Chi University, Hualien, Taiwan; 3 Institute of Plant and Microbial Biology, Academia Sinica, Taipei, Taiwan; 4 Institute of Bioinformatics and Systems Biology, National Chiao Tung University, Hsin-Chu, Taiwan; 5 Graduate Institute of Biotechnology, National Chung Hsing University, Taichung, Taiwan; 6 Department of Biotechnology, Ming Chuan University, Taoyuan, Taiwan; 7 Department of Biological Science and Technology, National Chiao Tung University, Hsin-Chu, Taiwan; East Carolina University, United States of America

## Abstract

**Background:**

Satellite RNAs (satRNAs), virus parasites, are exclusively associated with plant virus infection and have attracted much interest over the last 3 decades. Upon virus infection, virus-specific small interfering RNAs (vsiRNAs) are produced by dicer-like (DCL) endoribonucleases for anti-viral defense. The composition of vsiRNAs has been studied extensively; however, studies of satRNA-derived siRNAs (satsiRNAs) or siRNA profiles after satRNA co-infection are limited. Here, we report on the small RNA profiles associated with infection with Bamboo mosaic virus (BaMV) and its two satellite RNAs (satBaMVs) in *Nicotiana benthamiana* and *Arabidopsis thaliana*.

**Methodology/Principal Findings:**

Leaves of *N. benthamiana* or *A. thaliana* inoculated with water, BaMV alone or co-inoculated with interfering or noninterfering satBaMV were collected for RNA extraction, then large-scale Solexa sequencing. Up to about 20% of total siRNAs as BaMV-specific siRNAs were accumulated in highly susceptible *N. benthamiana* leaves inoculated with BaMV alone or co-inoculated with noninterfering satBaMV; however, only about 0.1% of vsiRNAs were produced in plants co-infected with interfering satBaMV. The abundant region of siRNA distribution along BaMV and satBaMV genomes differed by host but not by co-infection with satBaMV. Most of the BaMV and satBaMV siRNAs were 21 or 22 nt, of both (+) and (−) polarities; however, a higher proportion of 22-nt BaMV and satBaMV siRNAs were generated in *N. benthamiana* than in *A. thaliana*. Furthermore, the proportion of non-viral 24-nt siRNAs was greatly increased in *N. benthamiana* after virus infection.

**Conclusions/Significance:**

The overall composition of vsiRNAs and satsiRNAs in the infected plants reflect the combined action of virus, satRNA and different DCLs in host plants. Our findings suggest that the structure and/or sequence demands of various DCLs in different hosts may result in differential susceptibility to the same virus. DCL2 producing 24-nt siRNAs under biotic stresses may play a vital role in the antiviral mechanism in *N. benthamiana*.

## Introduction

RNA silencing is a sequence-specific gene regulatory mechanism involved in development, maintenance of genome integrity and cellular defense against virus infection in plants and animals [1], [2], [3], [4], [5]. Small interfering RNAs (siRNAs), diverse in size, sequence, biogenesis and biological actions, are the key mediators of RNA silencing [6]. In the RNA silencing process, the double-stranded RNAs (dsRNAs) or highly structured single-stranded RNAs (ssRNAs) introduced into a cell are recognized by the RNase-III enzyme Dicers and processed into siRNAs. These siRNAs are then recruited by ARGONAUTE (AGO) protein in RNA-induced silencing complexes (RISCs) and act in a sequence-specific manner to repressively regulate target gene expression by RNA degradation, translational inhibition or chromatin modification [7], [8].

The model plant *Arabidopsis thaliana* contains four DICER-like proteins (DCLs) faithfully generating size-specific siRNAs from both endogenous and exogenous dsRNA precursors [9]. DCL1 is mainly responsible for the biogenesis of endogenous microRNAs (miRNAs) [10]. DCL2 functions in the biogenesis of endogenous 24-nt natural antisense transcript-derived siRNAs (nat-siRNAs) induced by abiotic and biotic stress [11], [12]. DCL3 functions to process RNA-dependent RNA polymerase 2 (RDR2)-dependent dsRNA precursors to produce 24-nt siRNAs to direct DNA methylation [13]. DCL4 generates endogenous 21-nt *trans*-acting siRNAs (tasiRNAs) from RDR6-dependent dsRNA precursors [14], [15].

Despite DCLs processing endogenous precursors to siRNAs, all four DCLs are involved in producing the virus-derived siRNAs (vsiRNAs) during virus infection [16], [17], [18]. RNA virus infection usually results in elevated DCL4-dependent 21-nt and DCL2-dependent 22-nt vsiRNAs [16], [19], [20], [21], [22]. DCL4, DCL2 and DCL3 have specific, hierarchical antiviral activities against RNA viruses [16], [21]. DCL4 is the predominant sensor of RNA virus, whereas DCL2 has a subordinated anti-viral function and DCL3 a limited function. Although DCL3 can generate 24-nt vsiRNAs in RNA virus-infected plants when DCL2 and DCL4 are dysfunctional, DCL3 is unable to trigger antiviral silencing [21]. Also, DCL1 has no function in anti-RNA viruses [21]. However, siRNAs derived from DNA viruses could be generated by all DCLs [18].

Recently, genome-wide screening and analyses of siRNAs derived from *A. thaliana* were used with wild-type *Col-0* and several *dcl* and *rdr* mutants to diagnose the function of different DCLs and RDRs [23]. As well, the origin, processing and stability of siRNAs derived from viruses were studied in several DNA viruses, RNA viruses and viroids [18], [20], [21], [23], [24], [25], [26]; most of the vsiRNAs were shown to be positive-sense dominant [20], [25], [27]. In contrast, for satRNAs, only one study has investigated siRNAs derived from satRNAs associated with *Cucumber mosaic virus* satRNA (satCMV) [25]. Most of the siRNAs derived from satCMV showed positive-sense polarity and were produced from highly structured regions predominantly of 21 nt [25]. In addition, DCL4 was found to be the major enzyme for generation of these siRNAs [25]. However, all these reports focused on the viral or satRNA genome itself. The profile of vsiRNAs after satRNA co-infection has not yet been studied.

*Bamboo mosaic virus* (BaMV) belongs to the potexvirus genus and contains a single-stranded, positive-sense RNA genome that encodes five open reading frames (ORFs) [28]. BaMV-associated satRNA (satBaMV), the only example of satRNA associated with potexvirus, totally depends on BaMV for replication, assembly and movement and contains only one ORF [29]. P20, encoded by satBaMV, can be detected *in vivo* and *in vitro* [29], [30] but is not required for satBaMV replication or cell-to-cell movement [31]. It indeed facilitates satBaMV long-distance movement in *Nicotiana benthamiana* co-infected with BaMV [31], [32].

Previously, we characterized two representative satBaMVs: BSL6 and BSF4 [33]. The BSL6 is an interfering satBaMV that can greatly reduce BaMV accumulation and attenuate BaMV-induced symptoms in all tested plants, including *N. benthamiana* and *Chenopodium quinoa* [34], [35], [36], whereas BSF4, an non-interfering satBaMV, could not. BSL6 and BSF4 satBaMV share 93% nucleotide identity [33], but the key determinant of satBaMV-mediated interference of BaMV replication has been mapped to the apical hairpin stem loop (AHSL) located in the 5′ UTR of BSL6 [35]. One specific nucleotide change in the internal loop of AHSL could change the interference ability [36]. Nevertheless, the detailed mechanism has not yet been defined. Previously, Havelda et al. [37] reported that defective interfering RNA (DI-RNA) of *Tomato bushy stunt virus* (TBSV) could enhance the silencing pathway on elevating the level of TBSV vsiRNAs and result in saturation of the viral silencing suppressor and a high level of unbound vsiRNAs [37]. Such findings led us to investigate whether BSL6 satBaMV uses a similar strategy to interfere with replication of BaMV.

To analyze the profiles of viral and satellite siRNAs (satsiRNAs), we performed large-scale siRNA sequencing from BaMV-infected or satBaMV-co-infected plants followed by bioinformatics analyses. In plants co-infected with BaMV and BSL6, the level of siRNAs of BaMV was much less than that in plants infected with BaMV alone or co-infected with BSF4. In general, BaMV and satBaMV siRNAs are enriched in 21 and 22 nt of both strand polarity types and not evenly distributed in the viral or satRNA genome. The abundant region of siRNAs along BaMV and satBaMV genomes are altered in different infected hosts, which suggests that the DCL target site of the virus genome may be involved in the virus susceptibility of different hosts.

## Results

### Detection of small RNAs derived from BaMV or satBaMVs by northern hybridization

**Figure 1:**
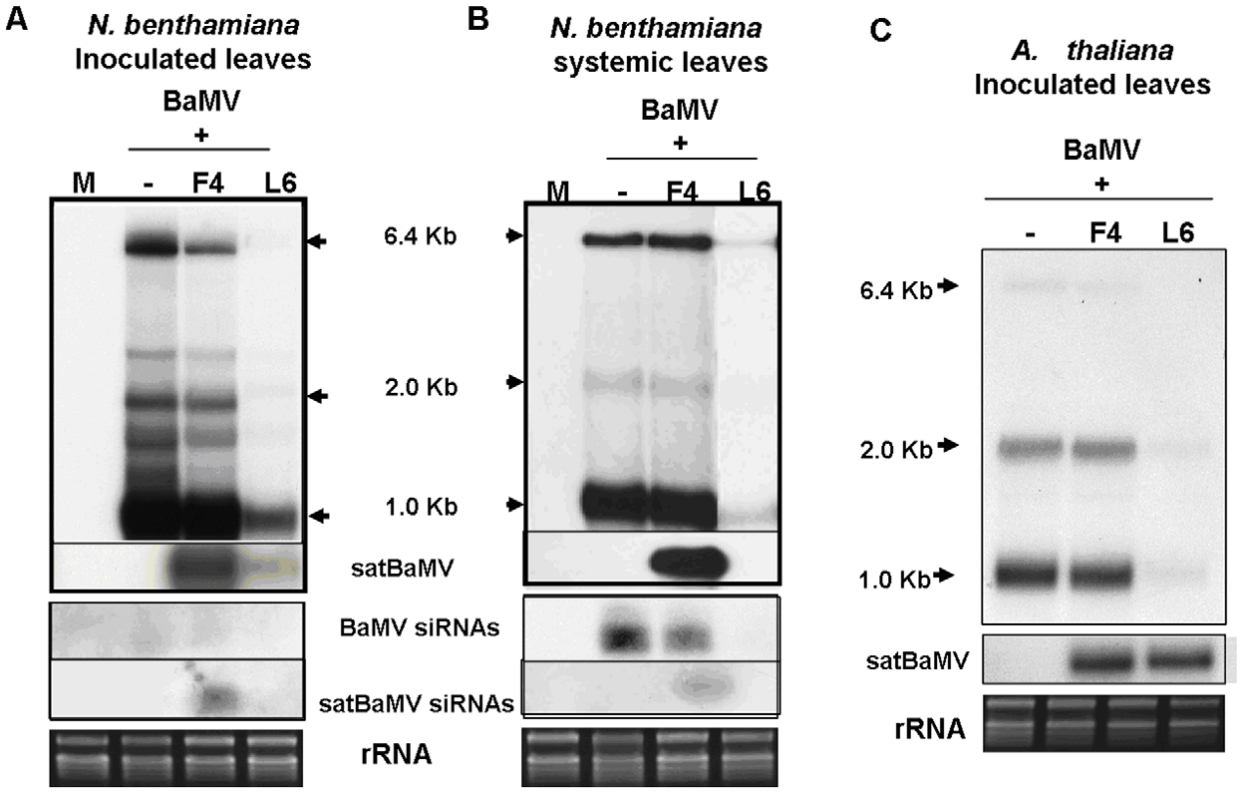
Accumulation of BaMV, satBaMV, and siRNAs in leaves of *N. benthamiana* and *A. thaliana*. Total RNA of 2.5 µg from *N. benthamiana* or 5 µg from *A. thaliana* were analyzed by northern blot analysis. The BaMV genomic (6.4 Kb), two subgenomic RNAs (2.0 and 1.0 Kb) and vsiRNAs were detected by a BaMV-specific probe and satRNA and satsiRNAs by a satBaMV-specific probe. M: mock (water) inoculation; −: BaMV alone. F4: noninterfering BSF4 satBaMV; L6: interfering BSL6 satBaMV. All films were exposed overnight except for detection of BaMV in *N. benthamiana* systemic leaves infected with BaMV or co-infected with BSF4 and BSL6 (3-hr exposure).

Viruses can induce post-transcriptional gene silencing (PTGS) as the antiviral mechanism in plants [1], [3], [38], [39], and subviral agents, such as viroids or satRNAs, can also trigger PTGS to produce small RNAs [40], [41]. *N. benthamiana* is highly susceptible to BaMV and satBaMV, which can move systemically and cause mosaic symptoms. To understand the association of the accumulation of BaMV and satBaMVs and their derived small RNAs, *N. benthamiana* was mechanically inoculated with BaMV and a noninterfering satBaMV, BSF4, or an interfering satBaMV, BSL6. The inoculated and systemic leaves were harvested at 8 and 20 days post-inoculation (dpi), respectively, for detection of the level of BaMV and satBaMV RNAs, vsiRNAs and satsiRNAs by northern blot analysis. As shown in Figure 1A and previously [35], [36], [42], BaMV accumulation was slightly reduced in the inoculated leaves of *N. benthamiana* on co-inoculation with non-interfering BSF4 satBaMV; however, BaMV accumulation was greatly reduced on co-inoculation with interfering BSL6 satBaMV. In BaMV-inoculated or BSF4 co-inoculated *N. benthamiana*, BaMV siRNAs were barely detected in the inoculated leaves, and BaMV siRNAs were not detected in the BSL6 co-inoculated leaves (Figure 1A). In contrast, a significant amount of BaMV siRNAs were detected, and the level was associated with the accumulation of BaMV, in the systemic leaves of BaMV-infected or BSF4-co-infected *N. benthamiana* (Figure 1B). The level of siRNAs derived from BSF4 was substantial in the co-inoculated and systemic leaves; however, neither BaMV nor satBaMV siRNAs could be detected in BaMV-infected and BSL6-co-infected inoculated or systemic leaves of *N. benthamiana* (Figures 1A and 1B), probably because of the low accumulation of both BaMV and satBaMV in the BSL6-co-inoculated plants. These results show that the levels of vsiRNAs and satsiRNAs are well associated with BaMV and satBaMV accumulation in inoculated and systemic leaves of infected *N. benthamiana*.

### Composition of the BaMV and satBaMV siRNAs in infected *N. benthamiana*

To study the profiles of siRNAs, total RNA extracted from inoculated and systemic leaves of *N. benthamiana* inoculated with water (mock), BaMV, BaMV and BSF4, or BaMV and BSL6 were submitted for Solexa sequencing.

After trimming the sequences, only the 17- to 28-nt siRNAs were further analyzed. More than 2 million siRNA sequences of 17 to 28 nt, including endogenous and virus- or satRNA-derived siRNAs, were obtained from each sample (Tables 1 and 2). In the BaMV-inoculated leaves, only 3.7% of total siRNAs were vsiRNAs, whereas in systemic leaves, vsiRNAs accumulated up to 17.5%. Co-inoculation with BSF4 did not change the trend. However, the amount of BSF4 siRNAs from inoculated leaves were about 3.5 times more than that from systemic leaves (5.1% in the inoculated leaves and 1.5% in the systemic leaves) (Table 1). In leaves with BSL6 co-inoculation, only a few siRNAs were matched to BaMV (0.1%) and BSL6 (0.2%), which corresponded with the low level of BaMV and BSL6 RNAs in inoculated leaves [35]. The level of vsiRNA (0.0%) and satsiRNA (0.0%) detected in the BSL6-co-inoculated systemic leaves was too low, and these data were excluded from further analysis (Table 1). All these results seem to be associated with the amount of viral or satBaMV RNAs that accumulated in leaves, which suggests that BSL6 may interfere with the replication of BaMV before gene silencing.

**Table 1:** The amount of small RNAs isolated from inoculated (I) and systemic (S) leaves of *Nicotiana benthamiana* inoculated with water (mock), BaMV alone or co-inoculated with BSF4 or BSL6.

	Mock	BaMV (I)	BaMV (S)	BaMV+BSF4 (I)	BaMV+BSF4 (S)	BaMV+BSL6 (I)	BaMV+BSL6 (S)
**Total**	2,489,506	3,293,585	3,411,741	5,247,031	3,567,466	3,272,499	4,853,059
**BaMV**		123,901 (3.7%)	596,851 (17.5%)	35,794 (0.7%)	829,730 (23.3%)	2,041 (0.1%)	95 (0.0%)
**BSF4**				269,101 (5.1%)	53,539 (1.5%)		
**BSL6**						7,441 (0.2%)	71 (0.0%)

**Table 2:** The polarity of small RNAs isolated from inoculated (I) and systemic (S) leaves of *Nicotiana benthamiana* inoculated with water (mock), BaMV alone or co-inoculated with BSF4 or BSL6.

	BaMV(I)	BaMV(S)	BaMV+BSF4(I)	BaMV+BSF4(S)	BaMV+BSL6(I)	BaMV+BSL6(S)
**BaMV (+)**	55,553	266,734	15,672	363,001	805	64
**BaMV (−)**	68,348	330,117	20,122	466,729	1,236	31
**B(+)/B(−)**	0.81	0.80	0.77	0.77	-	-
**satBaMV (+)**			115,896	24,488	2,678	62
**satBaMV (−)**			153,205	29,051	4,763	9
**satB(+)/satB(−)**			0.75	0.84	-	-

B: BaMV.

satB: satBaMV.

(+): positive strand of virus/satRNA genome.

(−): negative strand of virus/satRNA genome.

“-”: not determined.

We analyzed the polarity of virus-derived siRNAs and found negative-strand siRNAs of BaMV slightly higher in level than positive-strand siRNAs (about 0.8 ratio for positive-strand to negative-strand siRNAs) regardless of inoculated or systemic leaves, in all of the samples analyzed from *N. benthamiana*. A similar ratio of positive- to negative- strand satsiRNAs was observed (Table 2). No comparison for BSL6 siRNAs was performed because of the extremely low level (Table 2).

### Virus- and satRNA-derived siRNAs are mainly 21 and 22 nt, but endogenous 24-nt siRNAs are increased in level after BaMV and satBaMV infection in *N. benthamiana*

**Figure 2:**
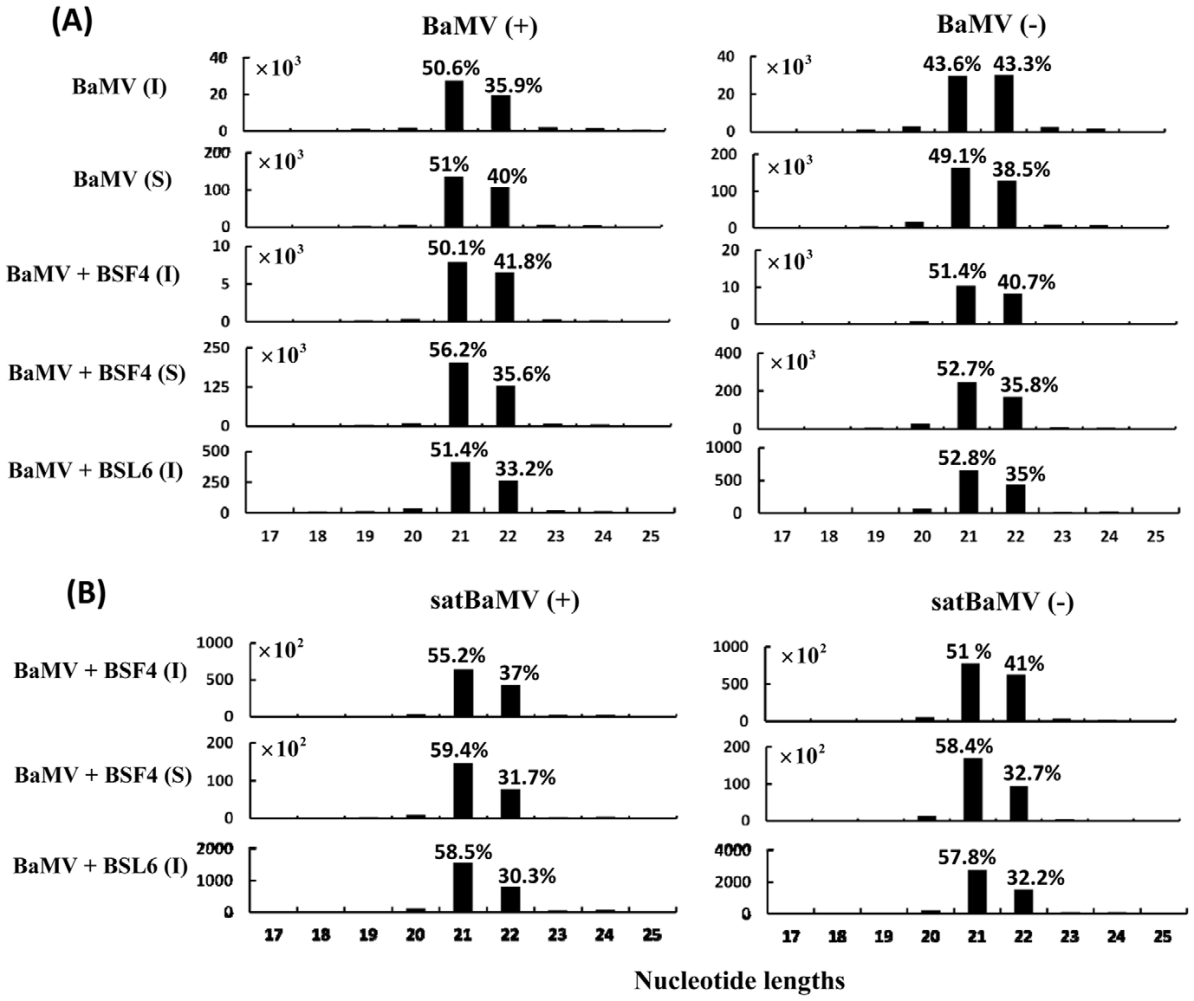
Size distribution of siRNAs derived from BaMV or satBaMV-co-inoculated *N. benthamiana*. The total siRNAs were isolated from BaMV-inoculated (I) and systemic (S) leaves, BaMV and BSF4 co-inoculated (I) and systemic (S) leaves, and BaMV and BSL6 inoculated leaves (I). (A) siRNAs matched to positive-strand (+) BaMV (left panel) or negative-strand (−) BaMV (right panel). (B) siRNAs matched to satBaMV (+) (left panel) or satBaMV (−) (right panel). Y axis represents the counts of siRNAs; X axis represents the length of siRNAs. The relative percentages of siRNAs of 21 and 22 nt to total siRNAs are shown above the bars.

With regard to size distribution, both positive- and negative-stranded siRNAs were predominantly 21 nt (43∼56%) and 22 nt (33∼43%), whether in inoculated or systemic leaves of BaMV-infected or satBaMV-coinfected *N. benthamiana* (Figure 2A). SatsiRNAs were also predominantly 21 nt (51∼59%) and 22 nt (30∼41%) in both positive and negative polarities in the inoculated and systemic leaves (Figure 2B). These results agree with previous reports of the hierarchical action of DCL4 and DCL2 in the production of most vsiRNAs and satsiRNAs of 21 and 22 nt.

**Figure 3:**
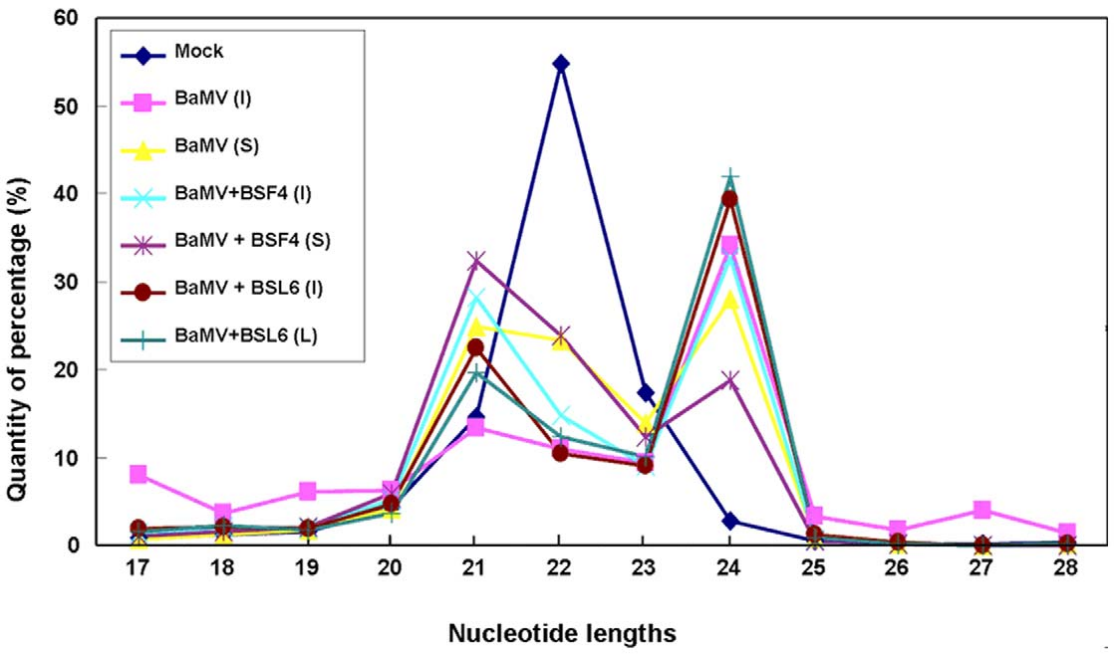
Size distribution of total small RNAs isolated from *N. benthamiana*. Total sRNAs ranging from 17 to 28 nt are shown on the X axis and relative percentages are shown on the Y axis. Small RNAs were collected from mock (⧫), BaMV-inoculated leaves (▪) and systemic leaves (▴), BaMV and BSF4 co-inoculated leaves (**×**) and systemic leaves (*), and BaMV and BSL6 co-inoculated leaves (•) and systemic leaves (**+**). Inoculated (I) and systemic (S) leaves were harvested at 8 dpi and 20 dpi, respectively.

To further analyze whether any bias in size distribution of siRNAs occurs in mock and virus-infected or satRNA-co-infected *N. benthamiana*, we compared the total siRNAs of 2–5 million reads. With mock inoculation, the sizes of siRNAs peaked at 22 nt, which represents the endogenous siRNAs. However, after BaMV inoculation or co-inoculation with satBaMVs, the profiles of total siRNAs changed. The population of 21-nt siRNAs increased and that of 22-nt siRNAs greatly decreased, whereas 24-nt siRNAs were induced to about 30%–40% of total siRNAs after BaMV inoculation and co-inoculation with satBaMVs, respectively (Figure 3). Because few 24-nt siRNAs were specific to BaMV or satBaMV (less than 1%) (Figure 2), most of the 24-nt siRNAs therefore represent the endogenous population. In *Brassica juncea*, the endogenous 21-nt siRNAs are the most abundant. After *Turnip mosaic virus* infection, 21- and 22-nt siRNAs were elevated to a higher level [43]. The same result was derived from *Cocksfoot streak potyvirus* (CSV)-infected *Dactylis glomerata* [44].

**Figure 4:**
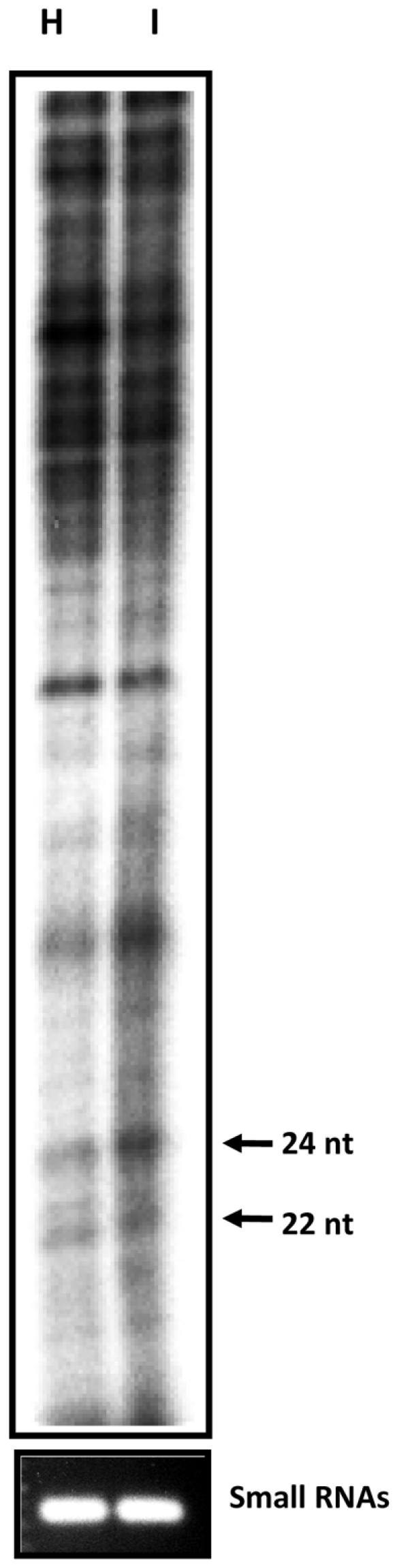
Comparison of the small RNAs between healthy and BaMV infected *N. benthamiana*. Small RNAs were isolated from healthy (H) or BaMV infected (I) leaves and 5′-end labeled with [γ-^32^P]-ATP. The 5′-end labeled small RNAs were separated by electrophoresis through a 10% acrylamide gel containing 7 M urea. The lower panel shows the quantity of equal loading by the separation of small RNAs on 1% agarose gel and stained with ethidium bromide. The positions of 22- and 24- nt RNAs are indicated by arrows.

To further confirm the shift in the size class of the most abundant endogenous small RNA species in host after BaMV infection, total RNAs were isolated from mock- and BaMV- infected *N. benthamiana*. The small RNAs were enriched and 5′-end labeled with [γ-^32^P]-ATP. The 5′-end labeled small RNAs separated by denaturing PAGE revealed that a stronger signal migrated at position of 24-nt RNAs in the BaMV-infected sample than that in the mock-inoculated control (Figure 4), which is consistent with the deep sequencing data.

### The abundant distribution of siRNAs along the BaMV and satBaMV genomes in *N. benthamiana*

**Figure 5:**
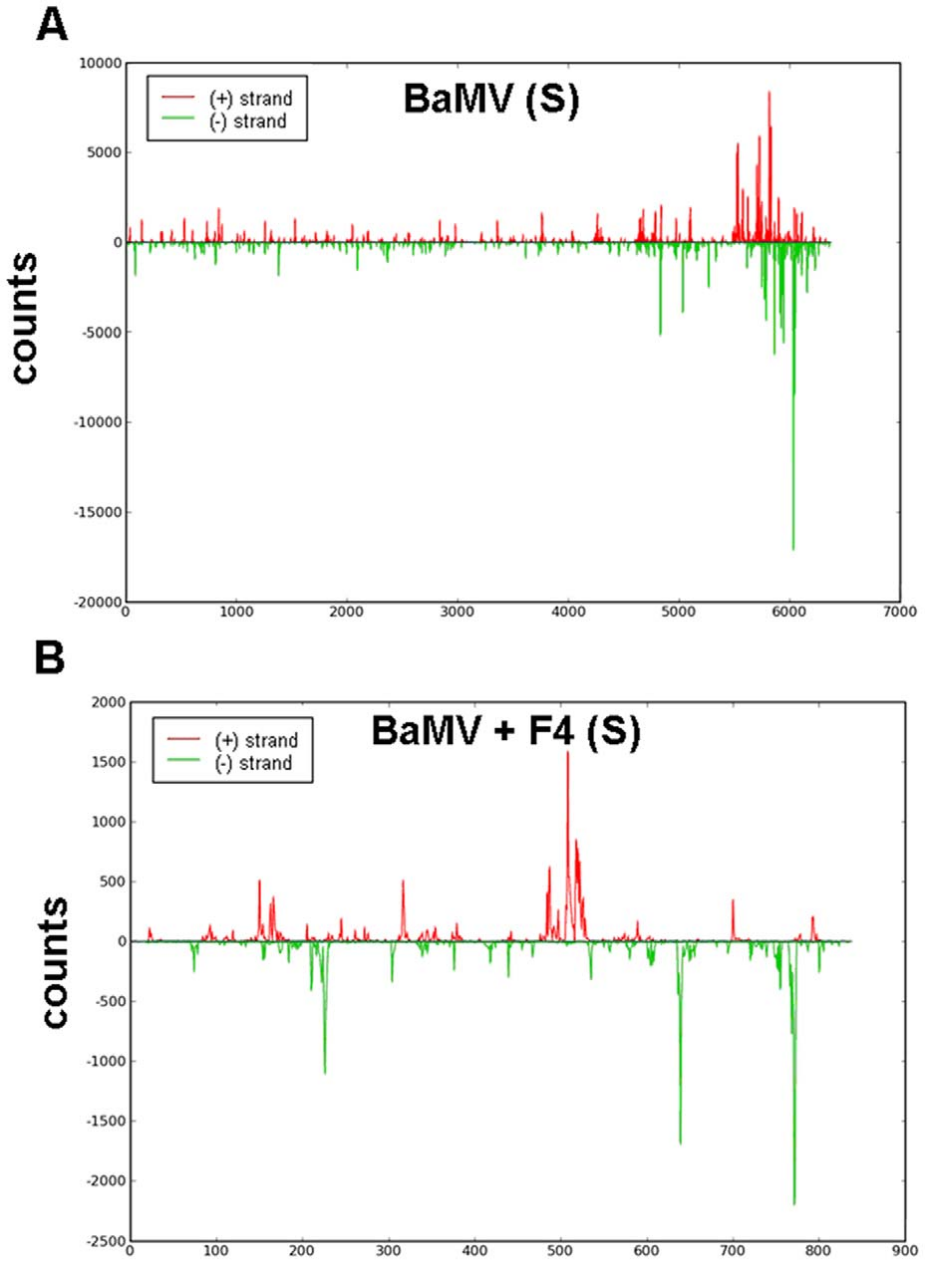
The abundant distribution of siRNAs on BaMV and satBaMV genomes from infected *N. benthamiana*. siRNAs derived from the viral genome of BaMV (A) and satBaMV (B) are shown in red above (positive strand) or green below (negative strand) the horizontal line. X axis represents the length of the genome, and Y axis the counts of the siRNAs.

To study the frequencies of vsiRNAs and satsiRNAs distributed in the BaMV and satBaMV genomes, respectively, we spotted all of the siRNAs, both positive- and negative-strand siRNAs, on the genomes of BaMV and satBaMVs (Figure 5). vsiRNAs exhibited similar patterns with or without satBaMV co-inoculation (Figures 5A and supplemental data 1A, S1A). The most abundant BaMV siRNAs were located within both positive and negative strands of the coat protein (CP)-coding region and 3′ UTR (Figures 5A and S1), possibly because of the high accumulation of CP-expressed subgenomic RNA in the leaves (Figures 1A and 1B) [29], [34]. However, only a few siRNAs were matched to BaMV or BSL6 satBaMV in the symptomless systemic leaves of BSL6-co-inoculated *N. benthamiana* (Tables 1 and 2).

Like BaMV siRNAs, BSF4 siRNAs showed similar patterns in both inoculated and systemic leaves (Figures 5B and S1B). The high abundant regions of satsiRNAs were in the positive strand of 500 to 550 nt and negative strands of 630 to 660 nt and 700 to 800 nt. Nevertheless, BSL6 siRNAs were more widely spread along the BSL6 genome, with some peaks, particularly in the negative strand of 200 to 250 nt and 600 to 660 nt (Figure S1B).

The major determinant of BSL6 in downregulating BaMV replication is located in the 5′ UTR, particularly in the hypervariable (HV) region, which folds a conserved AHSL structure [35]. We then mapped the 5′-end siRNA of two satBaMVs based on the secondary structures of 5′ UTRs [36], [45]. Both satsiRNAs peaked at the (−) strand of the HV region (Figure S2), which indicates the accessibility of target sites with AHSL. On analysis of satsiRNAs of BSL6, no BSL6-specific siRNA could be found to target the BaMV genome.

### Nucleotide preference of siRNAs from *N. benthamiana*

To investigate siRNA nucleotide preference, we first analyzed the GC percentage (GC %) of the virus-derived siRNAs. The GC % (mean 53.8%) of BaMV siRNAs was slightly higher than that of the BaMV genome (50.6%) in systemic leaves of BaMV-infected or BSF4-co-infected *N. benthamiana*. However, the GC % (mean 47.8%) of BaMV siRNAs in BaMV-inoculated leaves was lower than that of BaMV (Figure S3). Moreover, the GC % of satBaMV siRNAs (51.9%) was lower than that of the satBaMV genome (54.0%) in BaMV and BSF4-co-infected leaves. Next, we analyzed the internal stability at the 5′ end of the siRNAs. The positive-strand siRNAs derived from satBaMVs were AU rich at the first 7 nts (up to 59%), but this ratio was not obvious for the negative-strand satsiRNAs or those derived from BaMV (data not shown).

We then analyzed the 5′ terminal nucleotides of siRNAs. The bioinformatic data did not reveal any strong preference for the 5′ terminal nucleotide of BaMV- or satBaMV- specific siRNAs, whether derived from the (+) or (−) strand or were 21- or 22-nt siRNAs (data not shown). In summary, we found no nucleotide preference in the generation of BaMV and satBaMV siRNAs in infected *N. benthamiana*.

### The composition of BaMV and satBaMV siRNAs in *A. thaliana*

To compare the vsiRNAs and satsiRNAs in different plants, we challenged the model plant *A. thaliana*, a non-natural host of BaMV, with BaMV and satBaMVs. The inoculated and systemic leaves were harvested at 7 and 15 dpi, respectively. No visible symptoms were observed even after 1-month BaMV infection. However, BaMV RNAs could be detected in inoculated leaves but not in systemic leaves by northern blot analysis (Figure 1C and data not shown). Notably, BaMV level in inoculated leaves of BaMV-infected *A. thaliana* at 7 dpi was about one-fourth of that in inoculated leaves of BaMV-infected *N. benthamiana* at 8 dpi. These results indicated that BaMV caused symptomless infection and may not be able to move systemically in *A. thaliana*. Most importantly, BSL6-mediated interference with BaMV replication occurred in *A. thaliana* (Figure 1C), similar to that found in *N. benthamiana*. BSL6 satBaMV downregulated about 90% of BaMV accumulation in BSL6-co-inoculated *A. thaliana*, regardless of a similar level of BSF4 and BSL6 during co-infection. Hence, the inoculated leaves of BaMV-infected and BaMV- and satBaMV-co-infected *A. thaliana* were harvested at 7 dpi for deep sequencing.

Only a small proportion of sequenced small RNAs were derived from BaMV (0.7∼1.5%) or satBaMV (0.1%) in BaMV- and satBaMV-co-infected *A. thaliana* (Table 3) as compared with those in *N. benthamian*a (Table 1). This finding may be due to the low accumulation of BaMV and satBaMV in inoculated *A. thaliana*. Interestingly, in contrast to the negative-strand dominance of BaMV siRNAs found in *N. benthamiana*, the level of positive-strand vsiRNAs was nearly equal to or slightly higher than those of negative-strand vsiRNAs in *A. thaliana* (Table 4). The positive-strand satsiRNAs were also substantially greater in level than were negative-strand satsiRNAs, although the accumulation level of satsiRNAs was low.

**Table 3:** The amount of siRNAs isolated from inoculated *Arabidopsis thaliana* inoculated with water (mock), BaMV alone or co-inoculated with BSF4 or BSL6.

	Mock	BaMV	BaMV+BSF4	BaMV+BSL6
**Total**	4,676,816	3,437,925	2,037,033	2,221,999
**BaMV**		23,714 (0.7%)	29,880 (1.5%)	4,555 (0.2%)
**BSF4**			1,281(0.1%)	
**BSL6**				1,653 (0.1%)

**Table 4:** The polarity of siRNAs isolated from inoculated *Arabidopsis thaliana* inoculated with water (mock), BaMV alone or co-inoculated with BSF4 or BSL6.

	BaMV	BaMV+BSF4	BaMV+BSL6
**BaMV (+)**	11,937	15,763	2,725
**BaMV (−)**	11,777	14,117	1,830
**B(+)/B(−)**	1.01	1.16	-
**satBaMV (+)**		999	897
**satBaMV (−)**		282	756
**satB(+)/satB(−)**		-	-

B: BaMV.

satB: satBaMV.

(+): positive strand of virus/satRNA genome.

(−): negative strand of virus/satRNA genome.

“-”: not determined.

**Figure 6:**
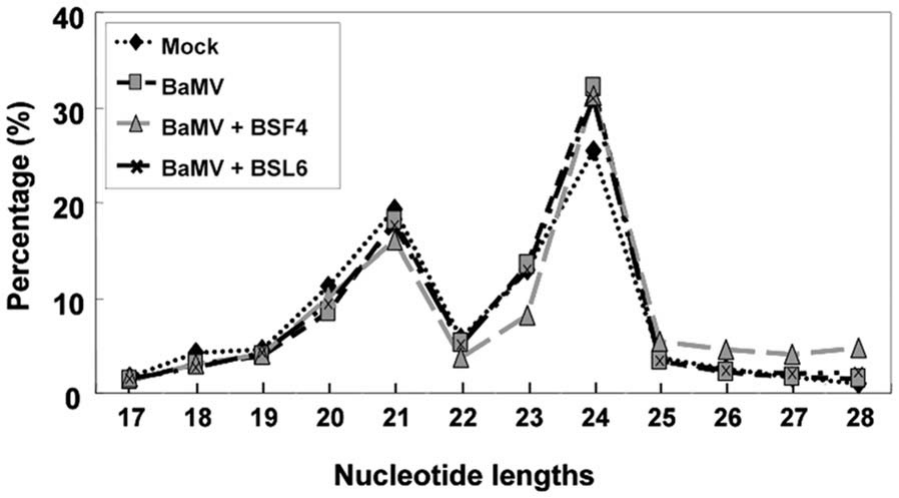
Size distribution of total small RNAs isolated from *A. thaliana*. Total sRNAs ranging from 17 to 28 nt are shown on the X axis, and relative percentages are shown on the Y axis. The percentages of sRNAs of different lengths of mock (♦), BaMV (■), and BaMV co-inoculated with BSF4 (▲) or BSL6 (**×**) plants are shown.

Analysis of the whole set of siRNAs, including the endogenous siRNAs, from *A. thaliana* revealed the 24-nt siRNAs the most predominant [23], and 21-nt siRNAs were the second highest in all treatments, including mock, BaMV, BaMV+BSF4 or +BSL6 infection (Figure 6). However, siRNAs matched to BaMV or satBaMVs were mainly 21 nt (Figure S4). This result differs from that of siRNAs from infected *N. benthamiana*. Most of the siRNAs isolated from mock-inoculated *N. benthamiana* were 22 nt but decreased in level after BaMV inoculation or co-inoculation with satBaMVs, which was followed by accumulation of the 24-nt siRNAs (Figure 3).

**Figure 7:**
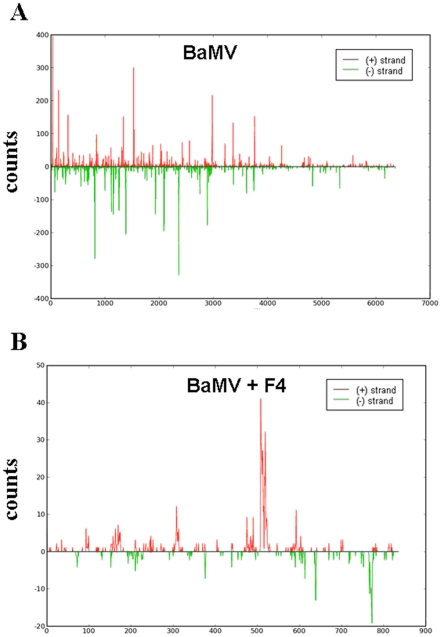
The distribution of siRNAs on the BaMV (A) and satBaMV (B) genome from *A. thaliana*. The siRNAs are shown in red above (positive strand) or in green below (negative strand) the horizontal line. X axis represents the length of the genome, and Y axis represents the counts of the siRNAs.

Regarding the distribution of vsiRNAs, most of the BaMV siRNAs from *A. thaliana* were derived from the first 5′ half of the genome with or without BSF4 co-inoculation (Figure 7 and data not shown). This finding is in contrast to results of vsiRNAs from *N. benthamiana* being largely generated from the CP and 3′ UTR regions (Figure 5). However, the distribution patterns of BSF4 siRNAs on the satBaMV genome were similar in *N. benthamiana* and *A. thaliana* (Figures 5B and 7B). In *A. thaliana*, the mean GC % of BaMV siRNAs (50.84%) was comparable to that of the viral genome (50.53%), but that of satBaMV siRNAs (49.81%) was a bit lower.

### Confirmation of the abundance and polarities of siRNAs derived from BaMV in *N. benthamiana* and *A. thaliana* by northern hybridization

**Figure 8:**
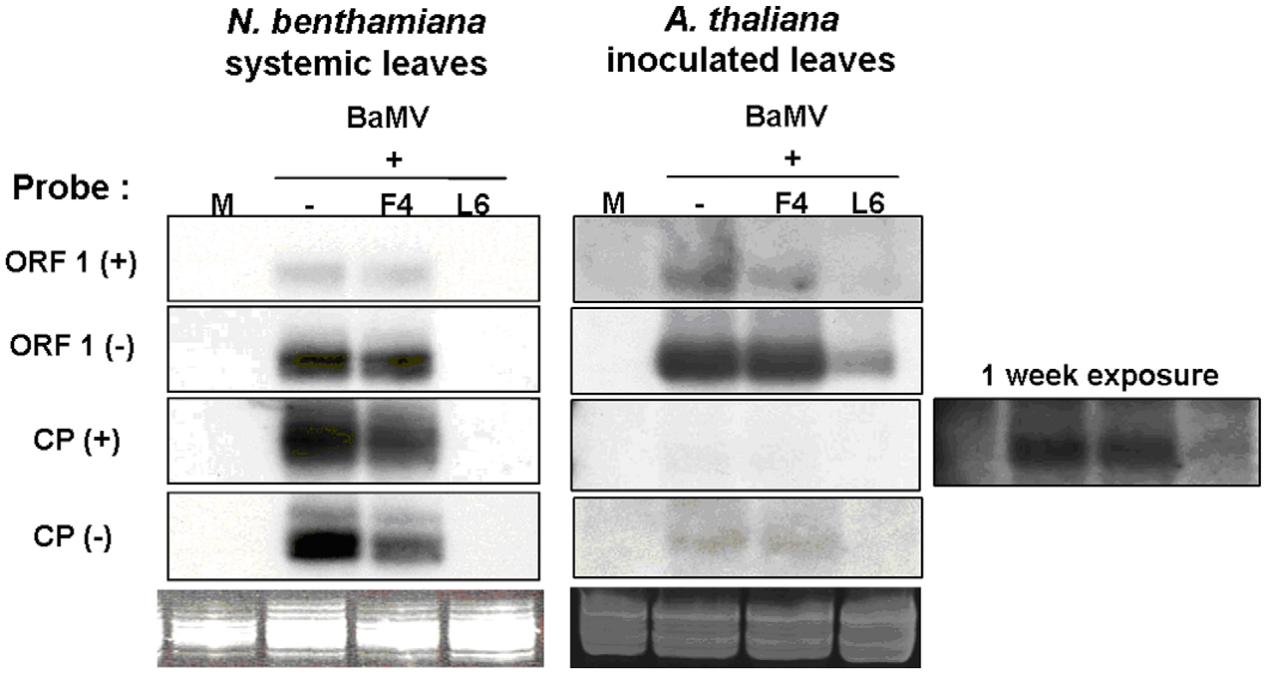
Northern blot analyses of siRNAs derived from BaMV in *N. benthamiana* and *A. thaliana*. Detection of BaMV siRNA to confirm the abundant regions in BaMV or satBaMV-co-inoculated *N. benthamiana* and *A. thaliana* by different probes as indicated. Total RNA of 25 µg from BaMV-inoculated or satBaMV-co-inoculated *N. benthamiana* or 45 µg from BaMV-inoculated or satBaMV-co-inoculated *A. thaliana* were loaded onto 19% acrylamide/7 M urea gel. The same blot was used for detection by different probes. All films were processed overnight, except *A. thaliana* probe with CP (+).

The abundant regions of siRNAs derived from BaMV were further confirmed by probes hybridizing to sense and anti-sense ORF1 and CP regions of the viral genome, respectively. In this experiment, RNA samples of BaMV-infected or BaMV- and satBaMV-co-infected systemic leaves of *N. benthamiana* at 20 dpi and inoculated leaves of *A. thaliana* at 7 dpi were hybridized with different probes by northern blot analyses. Although different probes may result in different intensities, the signals obtained in the northern hybridization (Figure 8) and the abundance of BaMV siRNAs (Figure 5) showed the same trend. In the systemic leaves of *N. benthamiana*, the CP probes showed stronger signals than did the ORF1 probes, and the CP (+) probe detected more than did the CP (−) probe, which indicates that vsiRNAs derived from the (−) strand of the CP region were indeed more abundant. All these results matched the findings for the siRNAs analyzed along the genome (Figure 5A, BaMV and BaMV+F4). As well, the CP probes detected fewer siRNAs than did the ORF1 probes in *A. thaliana* inoculated leaves. This finding agreed well with the deep sequencing results, whereby most of the BaMV siRNAs were located at the ORF1 region in *A. thaliana* (Figures 7 and 8).

### The DECLs generate similar cleavage sites in the BaMV/satBaMV genomes in the two different hosts

**Figure 9:**
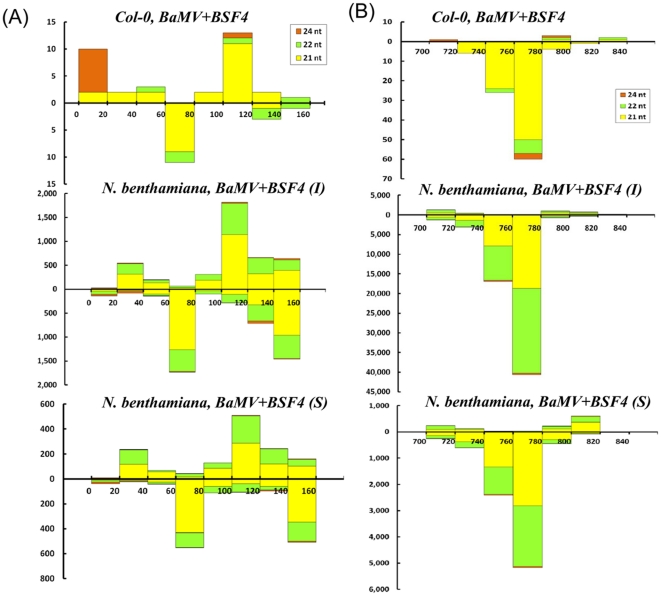
The distribution of siRNAs from sense and antisense BSF4 5′ (A) and 3′ (B) UTRs. The 21, 22 and 24 nt siRNAs derived from the genome of satBaMV BSF4 are shown in yellow, green and orange above (positive strand) or below (negative strand) the horizontal line, respectively. X axis represents the length of the genome, and Y axis represents the counts of the siRNAs.

Although the abundance of siRNA distribution along the BaMV genomes differed in the two infected plants: *N. benthamiana* and *A. thaliana*, refined analysis of the siRNA distribution revealed similar cleavage sites in BaMV or satBaMV, particularly in the 5′ or 3′ UTRs. For instance, in the 5′ UTR of BSF4, satsiRNAs peaked at the sense RNA nucleotides 100–120 in both *A. thaliana* and *N. benthamiana* co-infected with BaMV and BSF4 (Figure 9A). This peak is located within the internal loop II and short stem loop B [45]. Similarly, in the 3′ UTR of BSF4, satsiRNAs peaked at antisense RNA nucleotides 760–780 in both co-infected *N. benthamiana* and *A. thaliana* hosts (Figure 9B). This region resides between stem-loop B and stem-loop C (SLC) and one strand of stem region of SLC [46]. From secondary structural analysis of highly frequent regions within the 5′ and 3′ UTRs, the structural preference of DCLs could not be concluded. However, the predominantly produced 21-nt satsiRNAs in *A. thaliana* were replaced with an increasing amount of 22-nt siRNAs at the same cleavage site in both inoculated and systemic leaves of *N. benthamiana* (Figures 9A and 9B). This finding may account for the 40% of vsiRNAs and satsiRNAs being 22 nt in *N. benthamiana* but not in *A. thaliana* (Figures 2 and S4). These results also demonstrate that structure or sequence demands of different DCLs generating 21- and 22-nt siRNAs are similar in *N. benthamiana*.

## Discussion

In this study, we globally analyzed the siRNAs derived from BaMV and its non-interfering satRNA, BSF4, and interfering satRNA, BSL6, in two different plants, *N. benthamiana* and *A. thaliana*, by high-throughput sequencing followed by RNA hybridization analysis. In general, we obtained more than 2 million siRNA sequences from each sample by a combination of bioinformatic analyses, which provides detailed information about the interactions among viruses, associated satRNAs and hosts.

### Viral siRNAs in interfering satBaMV-co-infected plants

The interfering determinant region of BSL6 was previously mapped to the AHSL at the 5′ UTR of BSL6 [35], [36]. The mechanism of this satBaMV-mediated interference is still under investigation. Both DI-RNAs of *Cymbidium ringspot virus* (CymRSV) and TBSV can activate PTGS [37], [47], but the DI-RNA of CymRSV is a poor target of vsiRNAs [47]. As well, TBSV DI-RNAs elevated the vsiRNAs, thus saturating the virus silencing suppressor and increasing the amount of free-form siRNAs [37]. The 5′ AHSL of BSL6 may interfere with BaMV accumulation through a silencing mechanism. If so, we expected to detect greater vsiRNA and satsiRNA accumulation to target the viral genomes, followed by reduced accumulation of BaMV. However, analyses of the vsiRNAs and satsiRNAs in the BSL6-co-inoculated samples revealed the accumulation of only a few siRNAs in *N. benthamiana* (0.1% and 0.2%, respectively) and *A. thaliana* (0.2% and 0.1%, respectively) (Tables 1, 2, 3, and 4). The low abundance of vsiRNAs and satsiRNAs of BSL6 and its correlation with the accumulation of BaMV and satBaMV implies that the downregulation of BaMV by BSL6 occurs at the step before the production of siRNAs. In addition, we found similar BaMV small RNA profiles in size distribution and strand polarity with and without BSL6 co-infection (Figure 2). These results further suggest that the BSL6-mediated interference of BaMV accumulation may occur before the silencing event.

### The activity of hierarchy DCLs among different plant species

In the systemically infected host *N. benthamiana*, the siRNAs derived from BaMV or satBaMVs were mainly 21 and 22 nt (Figure 2), whereas those in non-systemically infected *A. thaliana* were 21 nt (Figure S4). A similar phenomena was observed in *Tobacco rattle virus* (TRV)-infected *N. benthamiana* and *A. thaliana* [24]. Further, the endogenous siRNAs from different plants were predominant in different sizes, for instance, 22 nt in *N. benthamiana*, 24 nt in *A. thaliana* [48] and 21 nt in *Brassica juncea* [43]. This finding indicates that the major DCL for producing the endogenous siRNAs is host dependent. Although most endogenous siRNAs were 22 nt in mock-inoculated *N. benthamiana*, the DCL4-dependent 21-nt siRNAs and DCL2- and DCL3-dependent 24-nt siRNAs were actively produced after virus infection (Figure 3). This finding agrees with previous reports that DCL4 is the major enzyme for generating vsiRNAs against virus infection in plants [16], [18], [21], [49] and DCL2 for the biogenesis of 24-nt nat-siRNAs under abiotic and biotic stress [12]. In the fungus *Cryphonectria parasitica* infected by a hypovirus, the expression of DCL2 for the generation of viral siRNAs was greatly induced [50]. This finding supports the notion that DCL enzymes could be induced after virus infection [50], but they were not commonly detected, probably because of the expression of a virus-encoded silencing suppressor [50] or because of the hierarchy and redundancy of DCLs in plants [21], [51].

Of note, 24-nt non-viral small RNAs were greatly enriched in number with virus infection (Figures 3 and 4). Among vsiRNAs produced in *N. benthamiana*, about 35–40% were 22-nt siRNAs. DCL2 may slice viral RNA directly into 22-nt siRNAs for antiviral activity and also regulate host gene expression by inducing 24-nt nat-siRNAs under biotic and abiotic stress, and to counteract virus offense. Because the whole genome sequence of *N. benthamiana* is unavailable, whether this large proportion of 24-nt small RNAs can target the host gene cannot be addressed. In addition, only about 15% of vsiRNAs were 22 nt, and the 24-nt non-viral small RNAs were not induced by BaMV infection in *A. thaliana* (Figures 5 and S4). This discrepancy in sizes of siRNAs in *N. benthamiana* versus *A. thaliana* could be due to far less accumulation of BaMV and satBaMVs in *A. thaliana* than in *N. benthamiana*, thereby generating fewer 22-nt vsiRNAs and satsiRNAs and corresponding 24-nt siRNA production. However, co-inoculation with non-interfering and interfering satRNAs would not alter the cleavage sites of BaMV viral RNA by silencing machinery (Figure S1A). Taken together, our results indicate that during virus infection or satRNA co-infection, the predominant siRNAs in *N. benthamiana* and *A. thaliana* differ in size. The activity of host-specific DCLs could be variable and dynamic, targeting the RNA sequence or structure preferentially in producing different siRNA profiles.

### Viral/satsiRNA asymmetry in strand polarity differs in different plants

Previously, most studies showed that cloned vsiRNAs originating from positive-strand RNA are more abundant than are those from negative-strand RNA, such as in *Turnip mosaic virus* [52], *Turnip crinkle virus* [27], [53], *Tobacco mosaic virus* (TMV) [54], *Potato virus X* (PVX) [20], CymRSV [20], CMV [52], satCMV [25] and *Peach latent mosaic viroid* (PLMVd) [55]. Also, the vsiRNAs of a DNA virus, *Cauliflower mosaic virus* (CaMV) [56], predominately originated from the translational leader with a secondary structure [56]. A few examples, such as vsiRNAs of *Cucumber yellow virus* and hypovirus-infecting fungi, showed an approximately equal ratio of positive- to negative-strand RNA [19], [50]. As well, only potato spindle tuber viroid (PSTVd) vsiRNAs are predominately derived from negative-strand RNA [57]. However, our results showed about 80% ratio of positive- to negative- strand siRNAs of BaMV in *N. benthamiana* infected with BaMV alone or with non-interfering or interfering satBaMV (Table 2); nevertheless, in *A. thaliana*, vsiRNAs of BaMV showed slightly higher abundance from positive-strand RNA (Table 4). The different asymmetry in strand polarity was also observed in TRV-infected *N. benthamiana* and *A. thaliana* [24]. This finding may be due to the accessibility of DCLs to various structural RNA molecules differing among hosts. Another possibility may be the different optimal temperature for growth of *N. benthamiana* and *A. thaliana*. The viral/sat RNA structure may fold differently under different temperatures and lead to the different viral/satsiRNA high peaks in these two different plants.

### Nucleotide preference of siRNAs

Regarding the selectivity of DCLs to the cutting site, internal stability at the 5′ end of the siRNAs plays an important role for incorporation into the RISC in studying the thermodynamics of functional siRNAs [58]. However, our GC % results of siRNAs or 5′ ends of siRNAs of BaMV/satBaMV could not show any bias in dicing activity (Figure S3). One explanation is that most of the siRNAs we analyzed but not those loaded into the RISCs were stable products after amplification and cleavage. This notion is supported by the study of TRV, in which a large proportion of TRV siRNAs were derived from processing viral dsRNAs generated by the action of endogenous RDRs or direct RDR products [24]. However, robust GC biases of TuMV and CSV siRNAs were observed in infected leaves of *B. juncea* and *D. glomerata* [43], [44]. The DICERs, which produce the vsiRNAs, could have preference for GC-rich regions, and this GC preference is considered an ancient feature of plant DCLs [44]. The controversial results infer that the GC preference of plant DCLs for viral RNAs remains debatable.

In terms of the the 5′ terminal nucleotide involved in sorting siRNA and thus guiding strands into AGO complexes, our data revealed no strong bias in siRNAs of BaMV/satBaMV. However, Donaire et al. [52] reported sense vsiRNAs enriched with a 5′-terminal U in plants infected with *Melon necrotic spot virus*, CymRSV, TRV, CMV, *Pepper mild mottle virus* and *Tomato yellow leaf curl virus*. However, plants infected with *Watermelon mosaic virus*, TuMV and PVX, showed equal proportions of sense vsiRNAs with a 5′ terminal U and A and antisense vsiRNAs with a 5′ terminal U, A or C [59]. Because sorting of siRNA guides strands into AGO complexes by the 5′ terminal nucleotide, data from Donaire et al. and our study suggested that vsiRNAs can be recruited into multiple AGO-containing complexes involving AGO1, which favors a 5′ terminal U; AGO2 and AGO4, which favor a 5′ terminal A; and AGO5, which favors a 5′ terminal C [60], [61]. Thus, different AGOs may play different roles in selecting sense and antisense vsiRNAs into RISC.

### The role of RNA silencing in the pathogenicity of viruses and satRNAs in different hosts

Delicate interactions among host, virus and satRNAs result in varied virus-induced symptoms. For satCMV, satsiRNAs with specific structural and conformational features play important biological roles in the regulation of host and helper-virus gene expression [25]. Host genes can be targeted and cleaved by TMV-derived siRNAs [54]. To this end, we predicted and scored host genes targeted by siRNAs of BaMV and satBaMV (both BSF4 and BSL6) (Figure S5) [54]. Some disease-related genes, such as several LRR family disease-resistance proteins and a GTP/RNA binding protein were the unique target of BSL6 siRNAs. Although these findings need to be further confirmed, interfering satBaMV may potentially manipulate the RNA interference machinery to generate BSL6-specific siRNAs that can target and regulate host genes and lead to downregulation of BaMV.

Surprisingly, the abundant distribution of BaMV siRNAs differed between *N. benthamiana* and *A. thaliana* but not on co-infection with BSF4 or BSL6 satBaMVs. The major target of DCLs resides within the BaMV genome region encoding the replicase in *A. thaliana* and the CP region in *N. benthamiana* (Figures 5A, 7A and 8). This finding may explain why *A. thaliana* is less susceptible to BaMV than is *N. benthamiana*. However, regardless of the different distribution of vsiRNA among the whole viral genome in different hosts, similar cleavage sites were found in the 5′ and 3′ UTRs of the BaMV and satBaMV genome (Figure 9). Although previous studies claimed that highly structured regions were more susceptible to DCLs [25], [56], the 5′ UTRs of BaMV and satBaMV are not the major spots of viral/satsiRNAs in both *N. benthamiana* and *A. thaliana* (Figures 5A, 5B, 7A, and 7B). The fewer viral/satsiRNAs generated from the 5′ UTR may be due to its less accessibility to DCLs by the complexity of proteins or protein–RNA or RNA–RNA interaction involved in replication and translation. Such stabilized RNA structures of 5′ and 3′ UTRs thus lead DCL to access the same cleavage sites among UTR regions in different hosts.

## Materials and Methods

### Plant materials and virus inoculation

*N. benthamiana* plants were grown in a growth chamber with a 16-h-light/8-h-dark cycle at 25°C. Three leaves of 1-month-old *N. benthamiana*, typically at the four-leaf stage, were used for inoculation, 4 plants for each experiment. Methods used for inoculation were previously reported [29], [31], except that inoculum contained 0.1 µg viral RNA extracted from BaMV-S virion derived from BaMV-S infectious clone pCB-infected *N. benthamiana* [62], [63] or the addition of 0.1 µg satBaMV transcript of pBSF4 [31] or pBSL6 [35]. Three inoculated leaves were harvested at 8 dpi, and the systemic leaves were harvested at 20 dpi for northern blot analysis, small RNA northern blot analysis and small RNA sequencing.

*A. thaliana* Col-0 seeds were sterilized and grown in solid MS medium for 8 days, then transferred to soil and grown in a growth chamber with 22/16°C day/night temperature cycles and a 16-h-light/8-h-dark cycle. Typically, for each set of experiments, 12 to 20 1-month-old *A. thaliana* plants were used for inoculation. Four to five rosette leaves of each plant were mechanically inoculated with BaMV-S viral RNA (0.1 µg/leaf) alone or co-inoculated with satBaMV transcripts (0.1 µg/leaf). All inoculated leaves were harvested at 7 dpi for northern blot analysis, small RNA northern blot analysis and small RNA sequencing.

### RNA isolation and northern blot analysis

Total RNA was extracted with use of TRI Reagent (Sigma-Aldrich, St. Louis, MO, USA) from 1 g inoculated leaves at 8 dpi and systemic leaves at 20 dpi from *N. benthamiana* plants or inoculated leaves at 7 dpi from *A. thaliana*. An amount of 2.5 µg total RNA of *N. benthamiana* or 5 µg total RNA of *A. thaliana* was used for BaMV and satBaMV detection by northern blot analysis.

For northern blot analysis, total RNA was denatured with use of glyoxal. Electrophoresis was conducted in 1% agarose gels. RNA was transferred to a Hybond™-N nylon membrane (GE Healthcare, Buckinghamshire, UK) by the capillary method with 3 M sodium chloride and 0.01 N NaOH and immobilized by UV cross-linking.

Hybridization was performed as described with probes labeled with [^32^P]CTP [31]. The ^32^P-labeled BaMV-specific probe, L probe, was prepared by linearization of pBaHB with *Hin*dIII, then transcription with SP6 RNA polymerase, which is complementary to the 3′ ends of positive-strand BaMV RNA [64]. The S probe, specific for the detection of positive-strand satBaMV RNA, was transcribed from *Eco*RI-linearized pBSHE by use of T7 RNA polymerase [31].

### Small RNA northern blot analysis

For BaMV and satBaMV small RNA detection, low-molecular-weight (LMW) RNA from 25 µg total RNA was enriched by 20% PEG-8000/3M NaCl. An equal volume of 50% deionised formamide with bromophenol blue was added to the LMW RNA and boiled at 95°C for 5 min, then placed on ice until being loaded into the 19% acrylamide/7 M urea gel. LMW RNA was transferred to a Hybond™-N nylon membrane by a semi-dry electroblotting system (Thermo Scientific Owl, New York, USA) and immobilized by UV cross-linking. Blots were prehybridized and hybridized at 42°C by use of ULTRAhyb®-Oligo (Ambion). The [^32^P]CTP-labeled ORF1 and CP probe were prepared as described previously [31], [64]. Briefly, BaMV 1 to 1024 nt was cloned into pGEMT-easy as an ORF1 probe. To reduce the size of the ORF1 and CP probes and to increase the hybridization efficiency for BaMV and satBaMV small RNAs, these probes were chemically hydrolyzed by use of alkaline carbonate buffer (8.5 mM Na_2_CO_3_ with 1 mM NaHCO_3_, pH 10.2) at 60°C for 1 hr 30 min before hybridization.

### Preparation of 5′-end labeled small RNAs

To label the 5′-end of the isolated total small RNAs, two µg of small RNAs were dephosphorylated with 2 units of alkaline phosphatase at 37°C for 1 h, followed by phenol/chloroform extraction, and then ethanol precipitation prior to kinase treatment [65]. The kinasing reaction was performed in a total volume of 20 µl containing 3 µl (10 Ci/µl) [γ-^32^P]-ATP and 5 units of T4 polynucleotide kinase, incubated at 37°C for 30 min, and then heat inactivated at 65°C for 10 min. The labeled small RNA were flowed into the G50 column (GE Healthcare, UK) to remove the free [γ-^32^P]-ATP. The 5′-end labeled small RNAs were resolved on 10% acrylamide gel containing 7 M urea. RNA markers with 23 and 21 nt were 5′-end labeled by the same method.

### Small RNA library sequencing

After total RNA was extracted from plants as indicated, small RNA libraries were generated following the manufacturer's protocol (Illumina, California, USA). Briefly, for each library, 10 µg of total RNA was size fractionated on a 15% tris-borate-EDTA urea polyacrylamide gel, and a 15–30 base-pair fraction was excised and purified. The 5′ RNA adapter (5′-GUUCAGAGUUCUACAGUCCGAC GAUC-3′) was first ligated to the RNA pool and a 40–60 base-pair fraction was gel purified. Then, the 3′ RNA adapter (5′- pUCGUAUGCCGUCUUCUGCUUGU-3′) was ligated to the isolated RNA, and the 60–100 base-pair fraction was excised and purified. Reverse-transcription PCR was used to create cDNA constructs based on the small RNAs with adapter molecules on both ends. The amplified PCR products were then gel purified. The purified PCR products were quantified on the Agilent DNA 1000 chip and sequencing was performed by use of an Illumina 1G Genome Analyzer (Illumina, California, USA).

### Bioinformatic analysis of small RNAs

The Illumina 1G sequencing reads were first trimmed to remove the adaptor sequence. The exact matches of the adapter sequence in the reads were identified in 3 steps as follows: 1) The adapter sequence was used as a probe, which allows for identifying exact match inserts. 2) If no adapter sequence was found, the last bases of the reads were probed successively with the first five bases of the adapter (minimum 5 bases) until a match was found, thus identifying inserts up to 30 bases. 3) Finally, the remaining reads were searched for no exact matches of the adapter. The first 4 bases of the adapter were used as a probe; the following bases were used to validate the adapter presence, and 70% of them had to be identical to the adapter sequence. The trimmed sequencing reads were then blasted to the BaMV (AF018156), BSF4 (AY205227) and BSL6 (AY205210) satBaMV, and only sequences with perfect matches were considered BaMV and satBaMV small RNAs.

## Supporting Information

Figure S1 The distribution of siRNAs on the BaMV (A) and satBaMV (B) genome from BaMV and BSF4- or BSL6-co-inoculated *N. benthamiana*. The siRNAs derived from positive-strand RNA (+) are shown in red above or negative-strand (−) in green below the horizontal line. The X axis represents the length of the genome, and the Y axis represents the counts of the siRNAs. S: systemic leaves. I: inoculated leaves.
(7.52 MB TIF)

Figure S2 The distribution of siRNAs in the 5′ UTRs of BSF4 (A) and BSL6 (B) satBaMV from BaMV and BSF4- or BSL6-co-inoculated *N. benthamiana*. The siRNAs derived from positive-strand RNA (+) are shown above or negative-strand RNA (−) below the horizontal line. The X axis represents the length of the 5′ UTR of satBaMV, and the Y axis represents the counts of the siRNAs. The secondary structures of 5′ UTRs of BSF4 and BSL6 satBaMV are shown in the right. The hypervariable (HV) regions folding into conserved apical hairpin stem loop (AHSL) are shown in red. S: systemic leaves. I: inoculated leaves.
(10.07 MB TIF)

Figure S3 The siRNA GC contents of BaMV (A) and satBaMV (B) genome from BaMV or BaMV and BSF4- or BSL6-co-inoculated *N. benthamiana* (Nb) and *A. thaliana* (At). The X axis represents different samples, and the Y axis represents the GC percentage of the siRNAs.
(7.22 MB TIF)

Figure S4 Size distribution of siRNAs derived from BaMV or satBaMV-co-inoculated *A. thaliana*. The total siRNAs were isolated from BaMV or BaMV and BSF4- or BSL6-co-inoculated leaves. (A) siRNAs matched to positive-strand (+) BaMV (left panel) or negative-strand (−) BaMV (right panel). (B) siRNAs matched to satBaMV (+) or satBaMV (−) genome. The X axis represents the length of siRNAs, and the Y axis represents the counts of siRNAs. The relative percentages of siRNAs of 21 and 22 nt to total siRNAs are shown above the bars.
(5.47 MB TIF)

Figure S5 Scores of host genes predicted as targets for BaMV in BaMV or BaMV- and satBaMV-co-inoculated *A. thaliana*. The X axis represents the counts of siRNAs and the Y axis represents the scores of the predicted host gene targeted by BaMV siRNAs.
(3.72 MB TIF)
