# Supplementary figures and images for: Global Analyses of Small Interfering RNAs Derived from Bamboo mosaic virus and Its Associated Satellite RNAs in Different Plants
Source: PLoS One. 2010 Aug 2;5(8):e11928. doi: 10.1371/journal.pone.0011928 (PMC2914070; doi:10.1371/journal.pone.0011928)

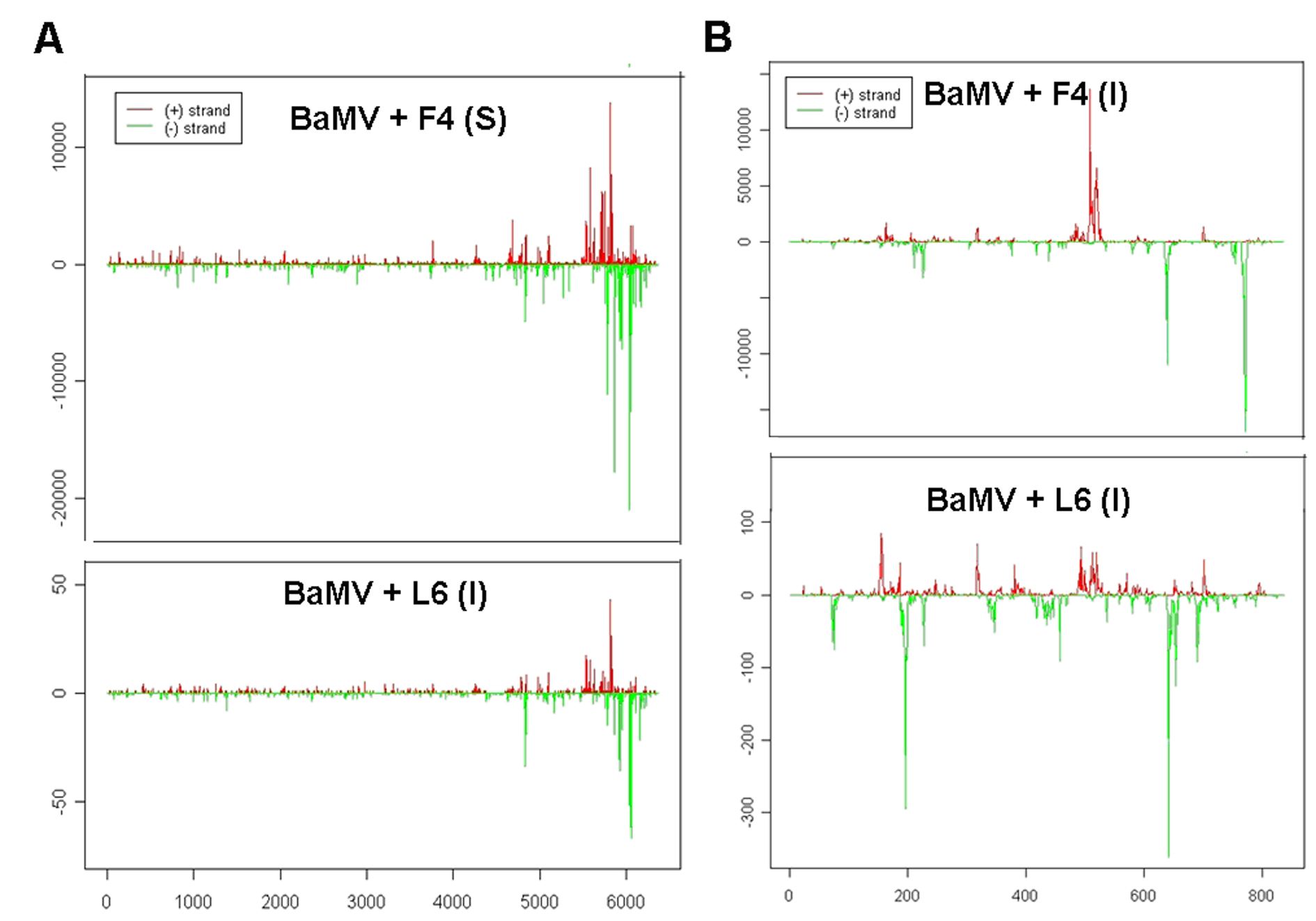

Supplement: Figure S1 — The distribution of siRNAs on the BaMV (A) and satBaMV (B) genome from BaMV and BSF4- or BSL6-co-inoculated N. benthamiana. The siRNAs derived from positive-strand RNA (+) are shown in red above or negative-strand (−) in green below the horizontal line. The X axis represents the length of the genome, and the Y axis represents the counts of the siRNAs. S: systemic leaves. I: inoculated leaves. (7.52 MB TIF) [file pone.0011928.s001.tif]

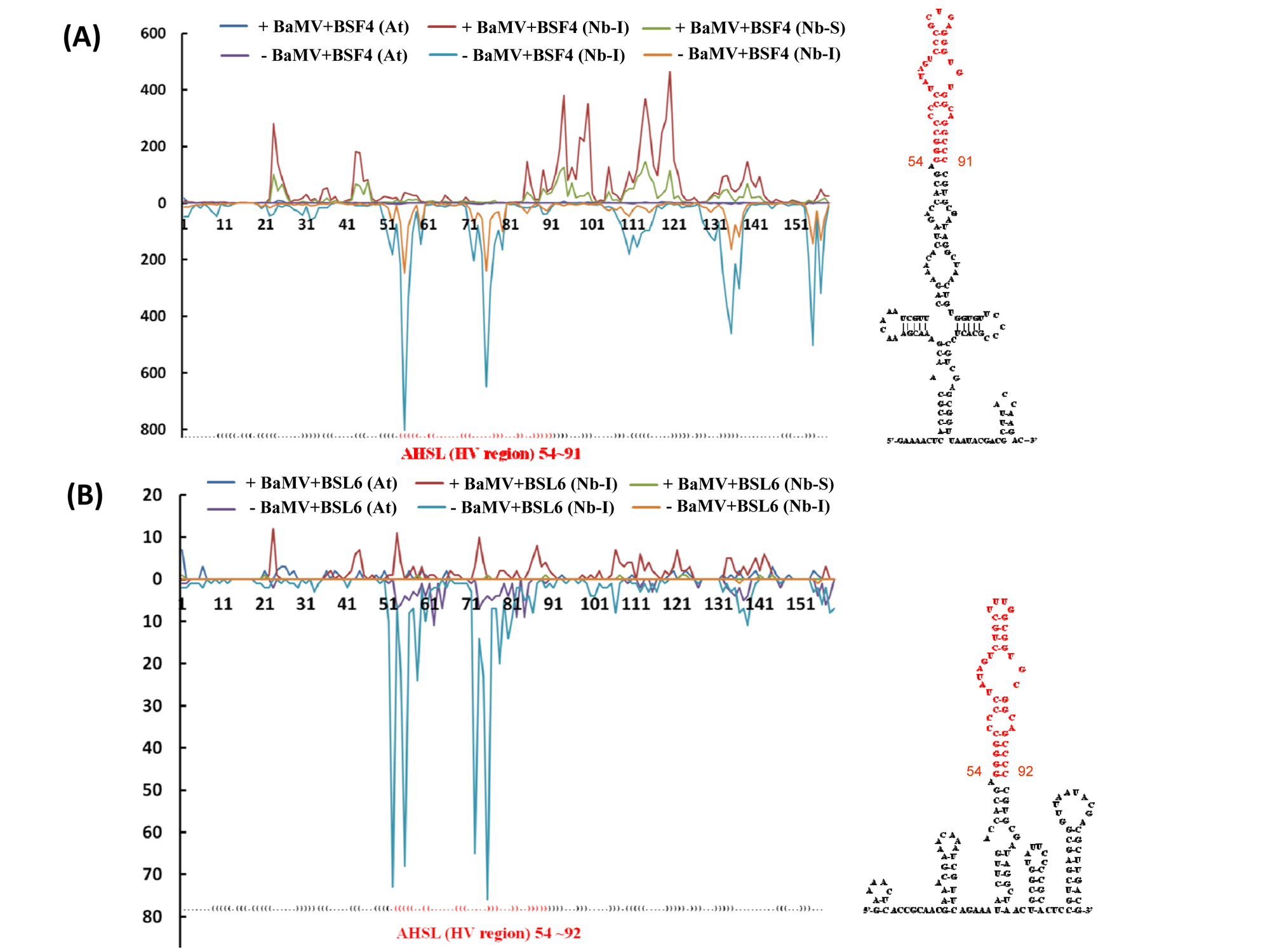

Supplement: Figure S2 — The distribution of siRNAs in the 5′ UTRs of BSF4 (A) and BSL6 (B) satBaMV from BaMV and BSF4- or BSL6-co-inoculated N. benthamiana. The siRNAs derived from positive-strand RNA (+) are shown above or negative-strand RNA (−) below the horizontal line. The X axis represents the length of the 5′ UTR of satBaMV, and the Y axis represents the counts of the siRNAs. The secondary structures of 5′ UTRs of BSF4 and BSL6 satBaMV are shown in the right. The hypervariable (HV) regions folding into conserved apical hairpin stem loop (AHSL) are shown in red. S: systemic leaves. I: inoculated leaves. (10.07 MB TIF) [file pone.0011928.s002.tif]

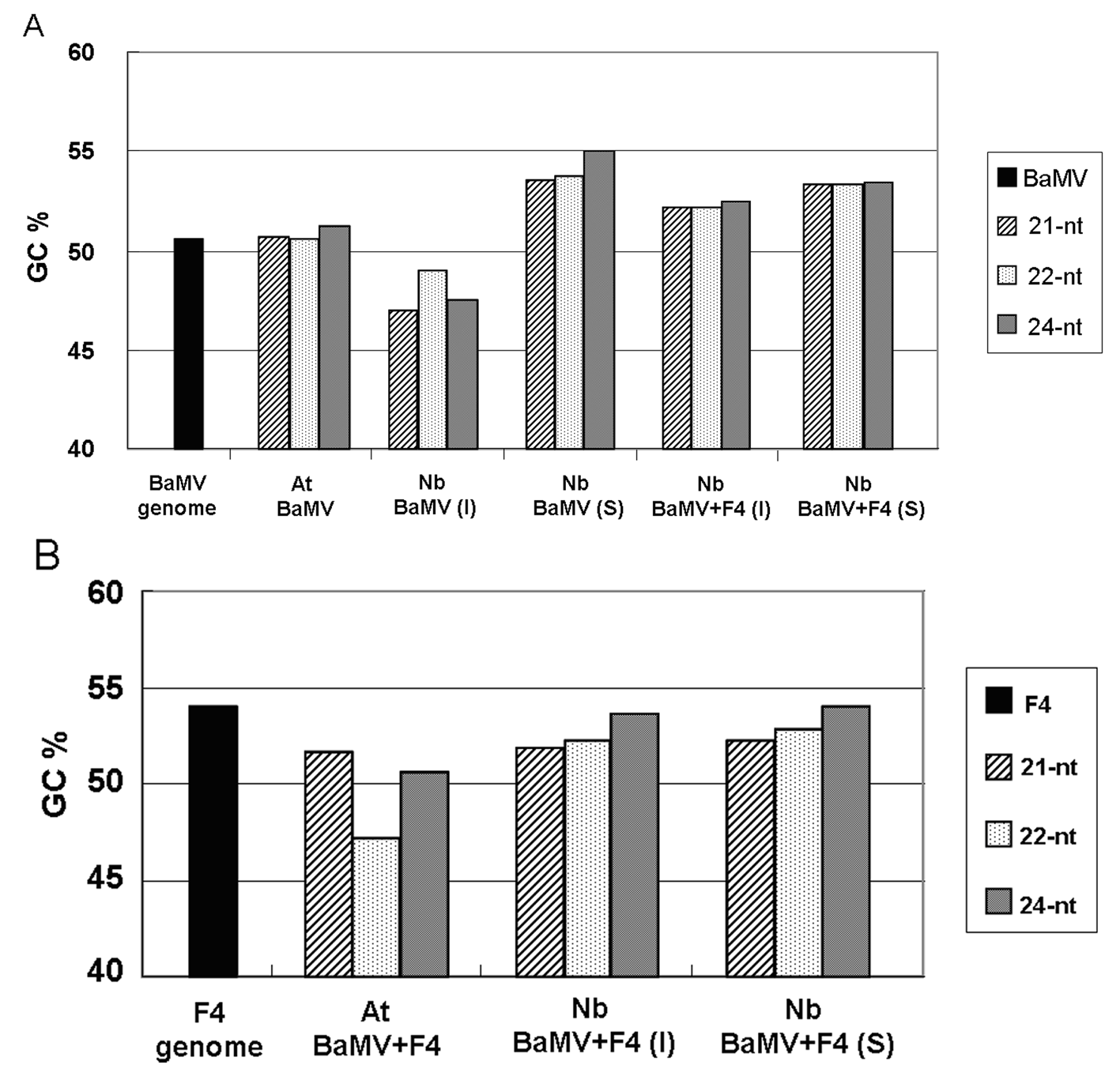

Supplement: Figure S3 — The siRNA GC contents of BaMV (A) and satBaMV (B) genome from BaMV or BaMV and BSF4- or BSL6-co-inoculated N. benthamiana (Nb) and A. thaliana (At). The X axis represents different samples, and the Y axis represents the GC percentage of the siRNAs. (7.22 MB TIF) [file pone.0011928.s003.tif]

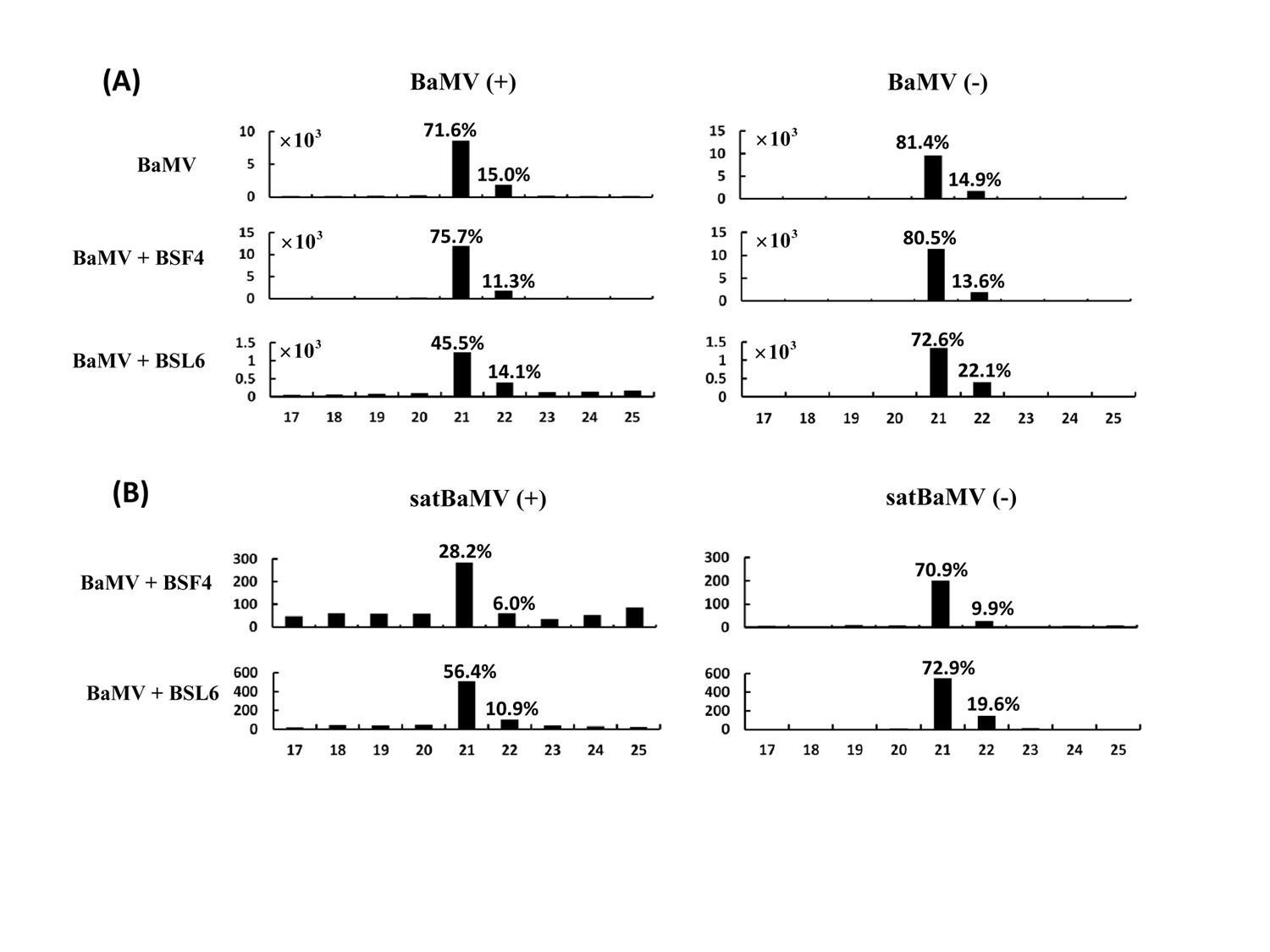

Supplement: Figure S4 — Size distribution of siRNAs derived from BaMV or satBaMV-co-inoculated A. thaliana. The total siRNAs were isolated from BaMV or BaMV and BSF4- or BSL6-co-inoculated leaves. (A) siRNAs matched to positive-strand (+) BaMV (left panel) or negative-strand (−) BaMV (right panel). (B) siRNAs matched to satBaMV (+) or satBaMV (−) genome. The X axis represents the length of siRNAs, and the Y axis represents the counts of siRNAs. The relative percentages of siRNAs of 21 and 22 nt to total siRNAs are shown above the bars. (5.47 MB TIF) [file pone.0011928.s004.tif]

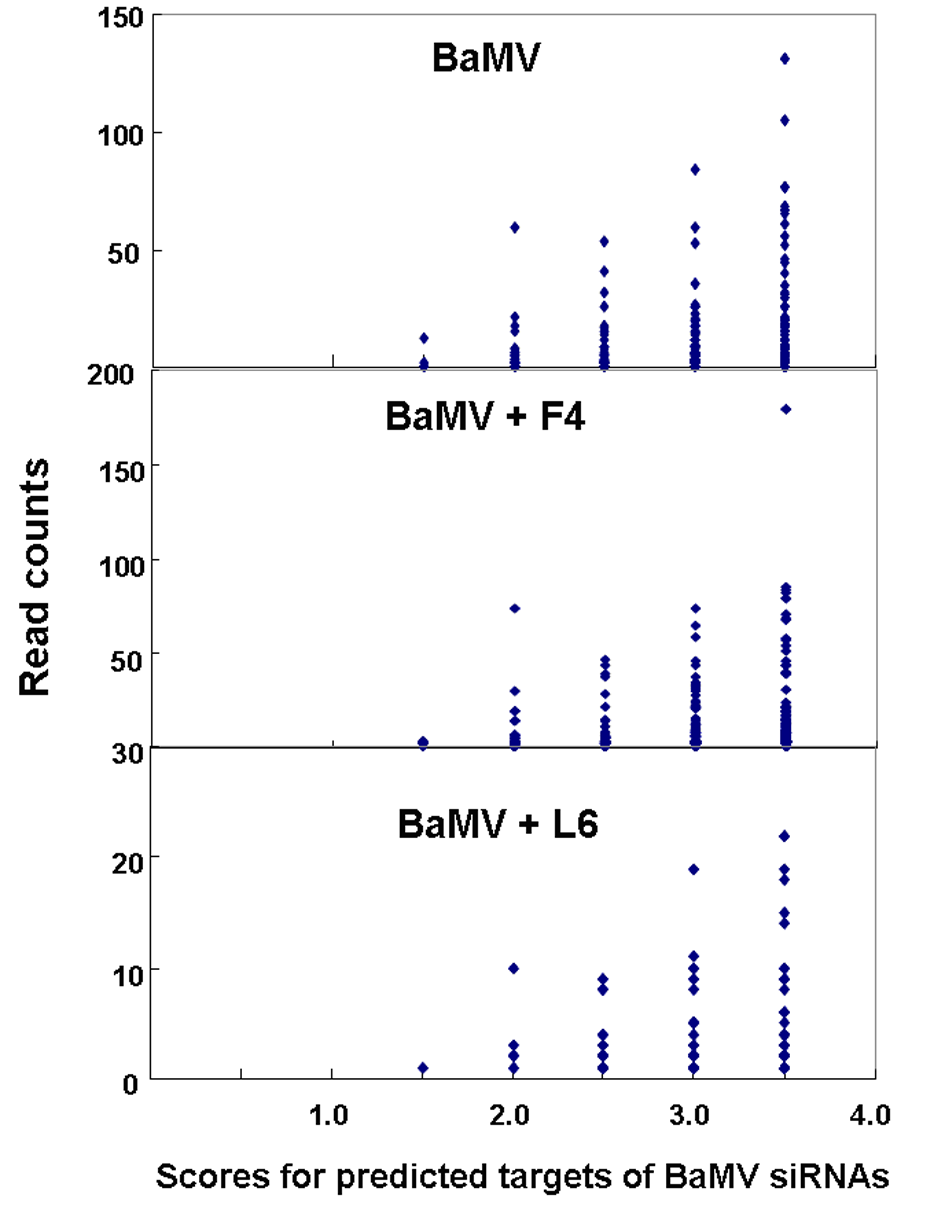

Supplement: Figure S5 — Scores of host genes predicted as targets for BaMV in BaMV or BaMV- and satBaMV-co-inoculated A. thaliana. The X axis represents the counts of siRNAs and the Y axis represents the scores of the predicted host gene targeted by BaMV siRNAs. (3.72 MB TIF) [file pone.0011928.s005.tif]
